# 

**Published:** 1854-05

**Authors:** 


*Hydrophobia.*—We notice that the very ridiculous story that ten deaths from hydrophobia, had been reported to the Common Council of Buffalo, by physicians, has found its way into the Boston Medical Journal. One death, or perhaps two, from hydrophobia, have occurred during the winter. There has been a good deal of alarm about it, and quite a number have been bitten by animals apparently rabid. In a very large proportion of the cases excision has been practiced. We hope that it may prove a sufficient safeguard against the horrible disease in question. H.

---

*Medical Men for Coroners.*—This is a measure of medical reform which must soon prevail. In Massachusetts it is already becoming the custom, and while it would not, perhaps, be worth while to make it a legal requirement, we hope to see the precedent established, and physicians appointed *to the office.* H.

---

"Types of Mankind," the new ethnological work by Nott, Gliddon, and Agassiz, has been received from the publishers, Lippincott, Grambo & Co. Its importance demands a careful examination, and we can only say for the present, that it is for sale by Leonard, 230 Main st.

---

Our next number will contain articles from Dr. Baker and from both the editors. We hope also to receive in time for our June issue, a letter from Dr. Flint, who has safely reached his transatlantic destination.

